# Impact of Salt Stress on Phytochemical Changes and Biological Activities of Quinoa Leaf Extracts In Vitro and In Silico

**DOI:** 10.3390/ph19050684

**Published:** 2026-04-27

**Authors:** Soumaya Arraouadi, Narmine Slimani, Hafedh Hajlaoui, Mabrouk Horchani, Karim Hosni, Antonio Cid Samamed, Mohamed Ali Borgi, Mejdi Snoussi

**Affiliations:** 1 Regional Center of Agricultural Research (CRRA) Sidi Bouzid, Gafsa Road Km 5, P.O. Box 357, Sidi Bouzid 9100, Tunisia; 2 Laboratory of Valorization of Unconventional Waters, INRGREF, University of Carthage, Road Hedi El Karray, El Menzah IV, PB 10, Ariana 2080, Tunisia; 3 Laboratory of Biotechnology and Biomonitoring of the Environment and Oasis Ecosystems (LBBEEO), Faculty of Sciences of Gafsa, University of Gafsa, Zarroug, Gafsa 2112, Tunisia; narmine.slimani96@gmail.com (N.S.); borgima@fsgf.ugaf.tn (M.A.B.); 4 Faculty of Sciences and Technology of Sidi Bouzid, University of Kairouan, Campus University Agricultural City, Sidi Bouzid 9100, Tunisia; bio.hafedh@gmail.com; 5 Laboratory of Plant-Soil-Environment Interactions, LR21ES01, Department of Biology, Faculty of Sciences of Tunis, University of Tunis EL Manar, Tunis 2092, Tunisia; 6 Laboratory of Heterocyclic Chemistry, Natural Products and Reactivity (LR11Es39), Chemistry Department, Medicinal Chemistry and Natural Products, Faculty of Science of Monastir, University of Monastir, Monastir 5000, Tunisia; horchani.mabrouk@gmail.com; 7 Laboratory of Natural Substances, National Institute for Research and Physico-Chemical Analysis, Sidi Thabet 2020, Tunisia; karim.hosni@inrap.rnrt.tn; 8 Departamento de Química Física, Facultade de Ciencias, Universidade de Vigo, Campus de As Lagoas s/n, 32004 Ourense, Spain; 9 Department of Biology, College of Science, Hail University, P.O. Box 2440, Ha’il 2440, Saudi Arabia; snmejdi@yahoo.fr

**Keywords:** quinoa, salt stress, HPLC-ESI-DAD-MS/MS profiling, antioxidant, antidiabetic activity, anti-obesity activity, in silico predictions

## Abstract

**Background:** This study investigated the effects of increasing levels of salinity on leaf phytochemical composition and the antioxidant, antidiabetic, and anti-obesity activities. **Method:** Three quinoa accessions grown under escalating NaCl treatments had their leaves exposed to various chemical analyses. Polyphenols, tannins, and flavonoids were among the phenolic substances whose concentrations were measured. The phenolic chemicals in the water extract were identified using HPLC-DAD-ESI-MS/MS. In vitro and in silico methods were used to measure anti-radical (DPPH), anti-alpha glucosidase, anti-alpha-amylase, and anti-lipase activities. **Results:** The results showed that water and ethanol, due to their polarity, were the most effective solvents for extracting phenolic compounds. Additionally, salt application led to a dose-dependent increase in total phenols (TPC), flavonoids (TFC), and tannins (CT) across all accessions. The accession DE-1 exhibited the highest contents with average values of 1453.03–4398.36 mg EGA/100 g DW, 322.7–1090.7 mg CAE/100 g DW, and 77.9–335.96 mg CAE/100 g DW of TPC, TFC, and Tannins, respectively. HPLC-ESI-DAD-MS/MS profiling of phenolic compounds led to identifying 18 constituents, including five major compounds (p-coumaric acid, caffeic acid, vanillic acid, p-coumaroyl hexose, and HHDP-galloyl glucose). Except for p-coumaroyl hexose and HHDP-galloyl glucose, which were extensively biosynthesized/accumulated in the salt-tolerant accession DE-1, the remaining phenolic compounds showed irregular evolution depending on accession and salt concentration. Moreover, ethanol and water extracts were evaluated for their anti-radical and enzyme-inhibitory activities. **Conclusion:** Salt-stressed DE-1 water extract showed strong antioxidant and enzyme inhibitory activities, indicating potential antidiabetic and anti-obesity effects. These activities were confirmed by in silico analysis.

## 1. Introduction

Climate change stands as one of the most critical global issues facing humanity in the 21st century [1]. It refers to long-term shifts in temperature and weather patterns, including intense drought episodes, water scarcity, rising temperatures, and changes in precipitation patterns [2]. Recent simulation models have demonstrated that climate change exerts a significant influence on soil properties, agricultural productivity, and biodiversity, particularly in arid and semi-arid regions. In these vulnerable regions, soil salinization is the most worrisome problem as it deeply influences the soil hydrodynamics, structure, salt movement patterns, microbial diversity, and crop water consumption [3,4].

In plants, increased soil and/or water salinity and its subsequent osmotic stress and ion toxicity affect all development and growth aspects [5]. The deleterious effects of salinity involve alterations in morphological (e.g., stunted growth, reduction in leaf area, chlorosis, senescence, inhibition of seed germination, decreased biomass production, low grain yield and weight, low seed number, and reduced inflorescence), physiological (e.g., reduction of chlorophyll content and inhibition of photosynthesis, stomatal conductance, decreased water content, nutrient imbalance, electrolyte leakage, and low osmotic potential) and biochemical (e.g., reduced C fixation, membrane damage, and oxidative stress) traits [6,7,8]. Despite the well-documented adaptive responses of plants to salinity, there remains insufficient focus on phytochemistry studies. One question that can be addressed is whether the changes in plants caused by salinity are subsequently followed by changes in their biological activities.

Plant metabolism (both primary and secondary) is particularly prone to climate change, having been shaped by the interaction of multiple stresses. While the general response includes a reduction in energy consumption and protein biosynthesis, an adaptive readjustment of the carbohydrate, amino acids, and polyamine metabolism has been observed in plants [9,10]. Additionally, a shift in secondary metabolism towards the biosynthesis of bioactive compounds involved in the acclimation of plants to combined stress has been reported [11,12,13,14]. The major metabolites induced by different stresses include polyphenols, terpenoids, alkaloids, carotenoids, etc. [15].

The use of tolerant species can overcome many current climate change-induced limitations. As such, quinoa (*Chenopodium quinoa* Willd.; family Amaranthaceae), a facultative halophyte native to the Andes highlands [16,17], emerges as a potential candidate for this purpose. Despite their high saponin content, quinoa seeds have garnered special attention for their nutritional value and health-promoting properties [18]. Quinoa leaves are often richer in various antioxidants, vitamins, and minerals, including protein, vitamin C, and carotenoids. It possesses a distinct nutritional profile characterized by elevated levels of nitrates and oxalates, making it a promising ingredient for functional foods. Like quinoa grains, the green leaves, sprouts, and microgreens of quinoa are also noted for their nutritional value and health-promoting properties. Numerous studies have documented the nutritional and bioactive components of quinoa leaves, highlighting their potential benefits in the diet [19,20,21].

Quinoa seeds and leaves are important sources of minerals, vitamins, proteins, fibers, lipids, saponins, phytosterols, terpenoids, and nitrogen-containing and phenolic compounds with intriguing food-related, nutraceutical, and functional properties [22,23]. In addition to its nutritional value, quinoa also possesses significant phytotherapeutic potential, including the reduction of the risk of cardiovascular diseases, neurodegenerative disorders, and diabetes [24]. Conversely, elevated glucose levels in diabetes mellitus activate various biochemical pathways that lead to the overproduction of reactive oxygen species (ROS), which typically play a role in regulating cellular signaling and homeostasis. As a result, phenolic compounds act as antioxidants, and dietary strategies that inhibit carbohydrate-metabolizing enzymes such as α-amylase and α-glucosidase can help reduce oxidative stress and improve metabolic control.

The content of quinoa metabolites and their subsequent nutritional and health-promoting effects are subject to variation depending on genetic factors (accession/variety), phenological stage, and abiotic stresses (salinity, alkalinity, drought, heavy metals, heat, etc.) [25,26]. A growing body of knowledge showed that quinoa can tolerate excessive salinity, reaching, in some cases, 750 mM NaCl [27]. Morphological, physiological, biochemical, and transcriptomic adaptations of quinoa to salt stress include, among others, reduction of leaf area, stomatal area, and density, the presence of periplasm, sequestration of Na^+^ in epidermal bladder cells and/or leaf vacuoles, improved K^+^ retention, a reduction of the activity of tonoplast channels, active H^+^ pumping in mesophyll cells, inactivation of photosynthesis, and accumulation of metabolites (e.g., proline, sugar, phenols, betaine, malate, cysteine, inositol, calcium oxalate, glutathione, and flavonoids). In addition, antioxidant enzyme activities, such as peroxidase (POX), superoxide dismutase (SOD), catalase (CAT), ascorbate peroxidase (APX), increased the up-regulation of salt overly sensitive (SOS) gene encoding the Na^+^/K^+^ antiport and the 9-cis-epoxycarotenoid dioxygenase (NCED) encoding for abscisic acid (ABA) [28,29].

Moreover, no study has directly linked salt-driven metabolomic reprogramming in quinoa leaves to inhibitory activities against key metabolic enzymes. Selecting accessions with contrasting salt-tolerance profiles (DE-1, 18 GR, and R-132) thus offers a unique framework to decode how stress adaptation intersects with the biosynthesis of bioactive compounds relevant to human health benefits such as type 2 diabetes and obesity management.

In this study, we examine the effects of quinoa leaf extracts on key enzymes—α-amylase, α-glucosidase, and lipase—that are closely linked to metabolic conditions leading to obesity and type 2 diabetes. α-Amylase initiates the digestion of starches by breaking them down into simpler sugars, and inhibiting this enzyme can slow starch digestion, thereby reducing the post-meal blood glucose peak. This reduction is crucial because elevated blood glucose levels contribute to insulin resistance, a hallmark of type 2 diabetes. Similarly, α-glucosidase further breaks down carbohydrates in the intestines, and its inhibition can also help manage glucose absorption, thereby supporting blood sugar control. Additionally, lipase plays a critical role in fat digestion by breaking down triglycerides into free fatty acids, influencing body weight and fat metabolism. By exploring the inhibitory properties of quinoa leaf extracts on these enzymes, we aim to highlight their potential as a therapeutic strategy for managing type 2 diabetes and obesity. Understanding the biochemical mechanisms of these reactions will provide valuable insights into the health benefits of quinoa and its phytochemicals. The present contribution was thus assumed to compare the salinity (NaCl) effect on phenolic leaf profiles of three quinoa accessions, and to evaluate their antioxidant, anti-obesity, and antidiabetic activities in vitro and in silico.

## 2. Results and Discussion

### 2.1. Phytochemical Profile: TPC, TFC, and CT

**Figure 1:**
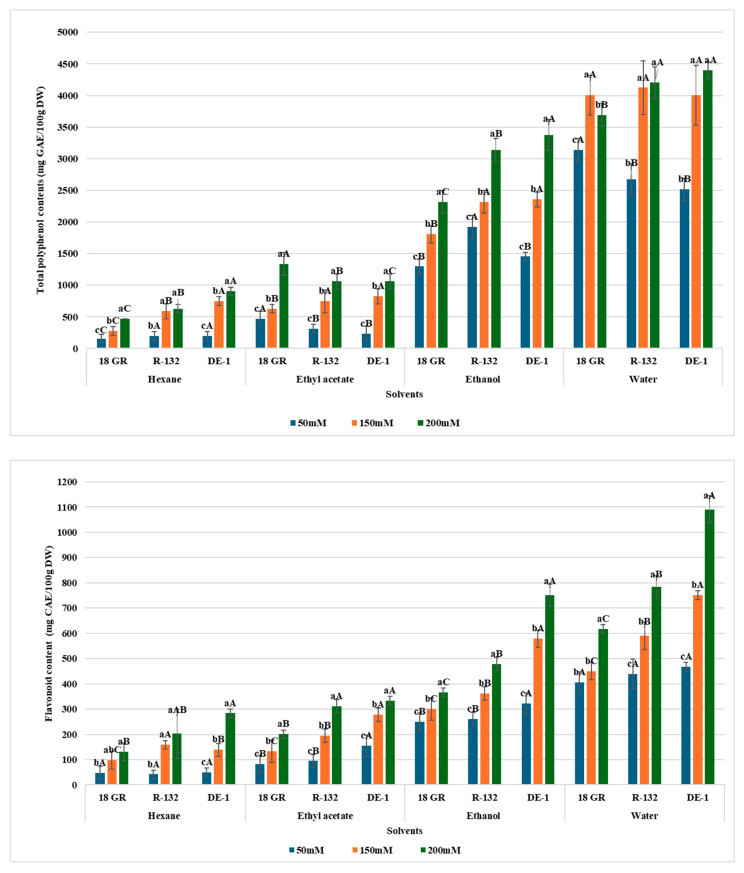

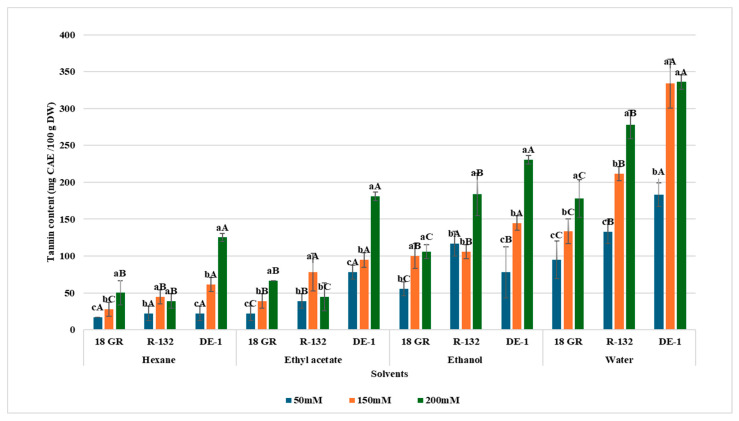
Concentrations of polyphenols, flavonoids, and tannins in leaf extracts of the three quinoa accessions under salt stress (50, 150, and 200 mM of NaCl corresponding to 0.29%, 0.88%, and 1.17%). Values are means ± SE (*n* = 3). Lowercase and uppercase letters (a, b, c and A, B, C) indicate mean comparisons between treatments and accessions, respectively, based on Duncan’s test. Means followed by the same letter are not significantly different (*p* < 0.05).

Preliminary phytochemical analysis showed that TPC, TFC, and CT contents decreased in the following order: water > ethanol > ethyl acetate > hexane (Figure 1). This finding indicates that polar compounds are particularly abundant in quinoa leaves. A comparison of the various accessions revealed that DE-1 exhibited the highest levels of TPC (1453.03–4398.36 mg GAE/100 g DW), TFC (322.7–1090.7 mg CAE/100 g DW), and CT (77.9–335.96 mg CAE/100 g DW). In addition to the accession factor, salinity significantly influenced the TPC, TFC, and CT contents (*p* < 0.05), suggesting its beneficial impact on the production and accumulation of these metabolites in salt-stressed quinoa. Owing to their antioxidant activity, the production of phenolics in response to salt stress could reflect an adaptive metabolic strategy recruited by quinoa to cope with the salinity-induced oxidative stress and its complications. The stimulatory effect of salinity on phenols/flavonoid production has also been reported for the Danish-bred quinoa (cv. Triticaca) [30], the ecotypes Salares R49, coastal-lowlands VI-1, and Villarica VR [31], and the varieties NSRCQE and NSRCQB [32]. Conversely, reciprocal trends have been observed in the salt-treated Danish cultivar Q52 [33] and quinoa accessions PECQ 20037, Negra Oruro, and Sajama [34].

It should be noted that the detection of low but non-zero levels of total phenolics, flavonoids, and tannins in hexane extracts may appear unexpected given the poor solubility of these compounds in non-polar solvents. This observation can be explained by several factors. First, minor carry-over of polar compounds during successive extraction steps may occur, particularly when solvents are applied sequentially. Second, certain lipophilic phenolic derivatives (e.g., methylated or prenylated forms) may exhibit partial solubility in non-polar solvents. Third, the Folin–Ciocalteu and related colorimetric assays are not entirely specific to phenolics and may react with other reducing substances such as terpenoids or carotenoids present in hexane extracts, leading to slight overestimation. Therefore, the values obtained for hexane extracts should be interpreted with caution. Similar limitations of solvent selectivity and assay specificity have been reported in previous studies [35,36,37].

The results of the present study suggest that accession, solvent, salt concentration, and their interaction are the major parameters determining the TPC, TFC, and CT contents, as they were cultivated, processed, and analyzed under the same conditions.

To evaluate whether the observed changes could affect their biological activities, polar extracts from the three quinoa accessions were tested for their in vitro antioxidant and enzyme-inhibitory effects.

### 2.2. In Vitro Bioactivity

**DPPH-radical scavenging activity**

As shown in Table 1, water extract exhibited the highest DPPH scavenging ability with IC50 values ranging from 6 to 30 µg/mL. For the samples of the same solvent, the highest anti-radical effect was observed for DE-1 leaf extract. Notably, irrespective of the solvent and accession, increased salinity significantly enhanced (*p* < 0.05) the DPPH-radical scavenging activity of quinoa leaves, as evidenced by the decreased IC50 values. The enhanced anti-radical activity may be associated with the increased TPC, TFC, and CT in response to elevated salinity. These results were consistent with those of previous studies that have linked the anti-radical activity of quinoa extracts to TPC and TFC [38,39]. However, other studies have demonstrated the absence of a clear relation between phenolic compounds (TPC and TFC) and the DPPH-radical scavenging activity, suggesting the involvement of different bioactive compounds, such as saponin, vitamin C, and carotenoids, in the antioxidant activity of quinoa [40,41]. The present study shows that high TPC, TFC, and CT could indicate higher anti-radical activity of quinoa leaf extracts.

**Enzyme-inhibitory effect**

Given that water extracts exhibited the highest DPPH scavenging ability (Table 2), it was chosen to evaluate their anti-lipase, anti-α-amylase, and anti-α-glucosidase activities.

The water extract was more effective against lipase than against α-amylase and α-glucosidase, and its enzyme-inhibitory activity was further exacerbated by increased salinity. For α-amylase, the enzyme hydrolyzes starch into reducing sugars, which react with DNS to form a colored complex; lower absorbance indicates fewer reducing sugars and therefore stronger inhibition. For α-glucosidase, the enzyme hydrolyzes p-nitrophenyl-α-D-glucopyranoside (pNPG) into p-nitrophenol; lower absorbance indicates higher inhibition. For lipase, the hydrolysis of 4-nitrophenyl caprate releases 4-nitrophenol, measured at 540 nm, where lower absorbance reflects stronger inhibition.

The significant enzyme-inhibitory effect of the water extract was associated with its high TPC, TFC, and CT, which is consistent with previous reports demonstrating the implication of phenolic compounds as potential inhibitors of pancreatic lipase, α-glucosidase, and α-amylase [42,43]. In previous studies, it was suggested that phenolic acids (e.g., ferulic, vanillic, p-coumaric acid, salicylic, 2-hydroxy-4-methylbenzoic, chlorogenic, etc.) and, to a lesser extent, flavonoids (e.g., rutin and quercetin) were responsible for the enzyme-inhibitory effects of quinoa seeds.

In the present study, the presence of putative phenolic compounds and/or other bioactive molecules (e.g., saponins, organic acids, etc.) with the ability to bind to the substrate, thereby limiting the fixation of enzymes to their substrates, could explain the enzyme-inhibitory effect of quinoa leaf extracts. The phenolic compounds of water extract were characterized by HPLC-PDA-ESI-MS/MS to confirm such a hypothesis, and its major compounds were studied in silico for their enzyme-inhibitory activity.

It is important to note that polyphenol-rich extracts, particularly those with high condensed tannin (CT) content, may induce non-specific enzyme inhibition through protein precipitation or aggregation. In the present study, although CT levels were markedly elevated in the DE-1 accession under 200 mM NaCl, the observed inhibition followed a consistent dose–response relationship across concentrations, supporting a mechanism predominantly based on specific enzyme–inhibitor interactions rather than non-specific effects. Nevertheless, the potential contribution of tannin-induced protein precipitation cannot be entirely excluded and should be considered a limitation. Future studies incorporating appropriate controls (e.g., polyethylene glycol (PEG)-mediated tannin precipitation assays) would help to further discriminate between specific and non-specific inhibition mechanisms [44].

### 2.3. HPLC-PDA-ESI-MS/MS Analysis of Phenolic Compounds of Water Extract

The phenolic profile of quinoa water leaf extracts is summarized in Table 3, along with their retention time, UV spectra, precursor ions, and MS2 fragments. A total of 18 compounds, including seven phenolic acids (p-coumaric, caffeic, vanillic, homovanilllic, 3-O-p-coumaroylquinic, p-coumaroyl hexose, and protocatechuic acids), five flavonols (quercetin-3-O-galactosylrhamnoside, quercetin-di-deoxyhexoside, rutin, kaempferol dihexoside, and quercetin hexoside), one flavone (myricetin pentoside hexoside), 1 flavanone (isosakunaretin pentoside rutinoside), one ellagitannin (HHDP-galloyl glucose), one aldehyde (dihydrobenzaldehyde glucuronide), and two organic acids (citric acid and methyl citrate) have been tentatively identified.

Salt treatment resulted in qualitative (disappearance of some compounds) and quantitative changes. As illustrated in Table 3, except for p-coumaroyl hexose and HHDP-galloyl glucose, which were extensively biosynthesized/accumulated in the tolerant DE-1 accession, the remaining phenolic compounds showed irregular evolution depending on accession and salt concentration. Some of the identified compounds are believed to be involved in the salt tolerance of quinoa by acting as direct quenchers of free radicals, owing to their high hydrogen donation ability. These include, but are not limited to, caffeic, protocatechuic, vanillic, and p-coumaric acids [45,46,47,48,49], rutin [47], quercetin hexoside [49], citric acid, and methyl citrate [50]. Regarding the bioactivity assays, the presence of some identified components such as rutin [51], vanillic acid, p-coumaric acid [52], procatechuic acid, and glycosylated forms of quercetin, kaempferol, and isosakunaretin [53,54], citric acid [55], and HHDP-galloyl glucose [56], among others, has been associated with antioxidant activity and inhibition of lipase, α-amylase, and α-glucosidase activities of quinoa leaves and seeds.

A comparative analysis with previously published studies on quinoa seeds under salinity stress reveals notable organ-specific differences in phenolic composition. In the present study, quinoa leaves—particularly from the salt-tolerant DE-1 accession—were characterized by a marked accumulation of ellagitannins such as HHDP-galloyl glucose and glycosylated phenolic acids such as p-coumaroyl hexose. In contrast, earlier reports on quinoa seeds subjected to saline conditions predominantly describe the accumulation of simple phenolic acids (e.g., ferulic, vanillic, and p-coumaric acids) and flavonoids such as rutin and quercetin derivatives [31,33,42]. These differences suggest a clear organ-specific metabolic reprogramming in response to salt stress, where leaves and seeds mobilize distinct branches of secondary metabolism. While seeds appear to preferentially activate the shikimate–phenylpropanoid–flavonoid pathway leading to flavonol accumulation, leaves may enhance the biosynthesis of hydrolyzable tannins, including ellagitannins, which are known for their strong antioxidant and metal-chelating properties. Such organotropic specialization likely reflects the distinct physiological roles of these tissues, with leaves requiring rapid and efficient protection against oxidative stress induced by salinity, whereas seeds prioritize storage-compatible and protective phenolics. This functional divergence provides new insight into the complexity of quinoa’s adaptive metabolism under saline environments and highlights the importance of considering tissue-specific responses when evaluating stress-induced phytochemical changes [27,28,29].

### 2.4. Molecular Docking Analysis

The prediction of the molecular docking properties was performed focusing on five major phenolic compounds (C1: p-coumaric acid, C2: caffeic acid, C3: vanillic acid, C4: p-coumaroyl hexose, and C5: HHDP-galloyl glucose) that could be responsible for the biological efficacy obtained in vitro. As shown in Table 4, HHDP-galloyl glucose exhibited binding energies of −10.5, −9.5, and −9.8 kcal/mol, respectively, when docked with α-amylase (3baj), α-glucosidase (8yie), and lipase (1ys1), indicating its strong in silico binding affinity (surpassing that of the standards, acarbose and orlistat). Docking scores are expressed as binding energies in kcal/mol, with more negative values indicating stronger predicted binding between the compound and the enzyme’s active site. This is consistent with the findings of previous studies, which reported that the enzyme-inhibitory effects of HHDP-galloyl glucose were mediated through non-specific interactions with the hydrophobic side chains of amino acids and/or hydrogen bonds between phenolic groups and polar groups of proteins, leading to its aggregation [56].

**Figure 2:**
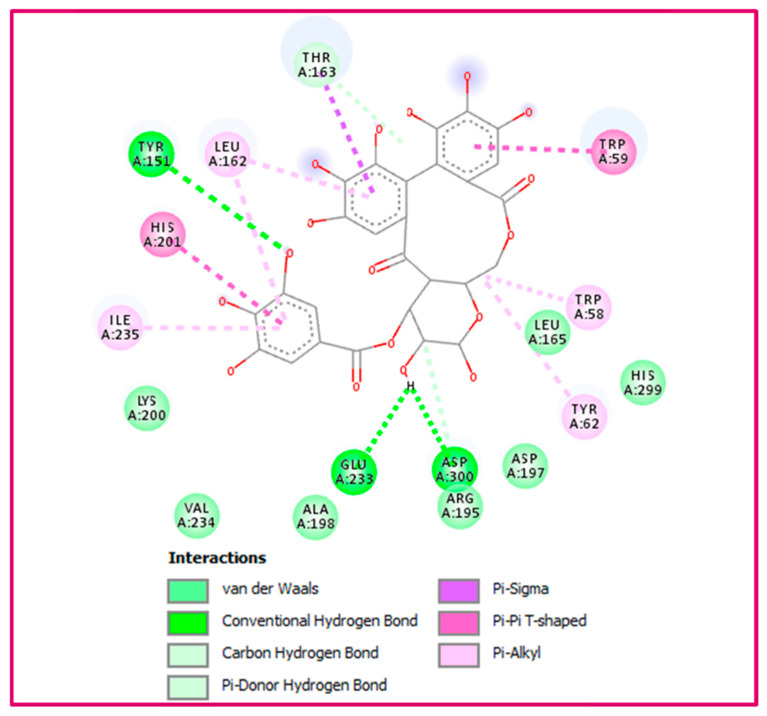
Shows how the most active molecule, Hhdp-galloyl glucose, binds to alpha-amylase’s binding cavity (PDB:3baj).

The confirmation of these results is given in Figure 2, showing that this ligand forms a more stable docking complex with α-amylase by establishing three conventional hydrogen bonds through its hydroxyl groups with Tyr151, Glu233, and Asp300. In addition to this, a number of other contacts, such as carbon–hydrogen bond (Arg195), Pi–donor hydrogen bond (Thr163), Pi–sigma (Thr163), Pi–Pi T-shaped (Trp59 and His201), as well as Pi–alkyl (Trp58, Tyr62, Leu162, and Ile235).

**Figure 3:**
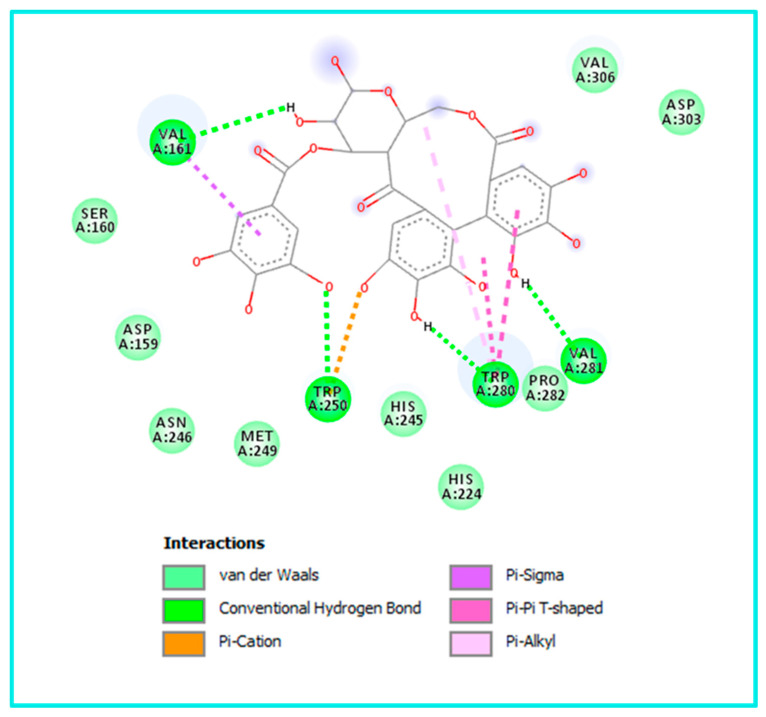
Alpha-glucosidase’s binding cavity contains the most active molecule, HHDP-galloyl glucose (PDB: 8yie).

Similarly, the HHDP-galloyl glucose interacted with the α-glucosidase enzyme by forming four H-bonds with Val161, Trp250, Trp280, and Val281. Additionally, some other interactions were observed, including Pi–cation with Trp250, Pi–sigma with Val161, and Pi–Pi T-shaped with Trp280. The latter amino acid is implicated in the Pi–alkyl interaction, as shown in Figure 3.

**Figure 4:**
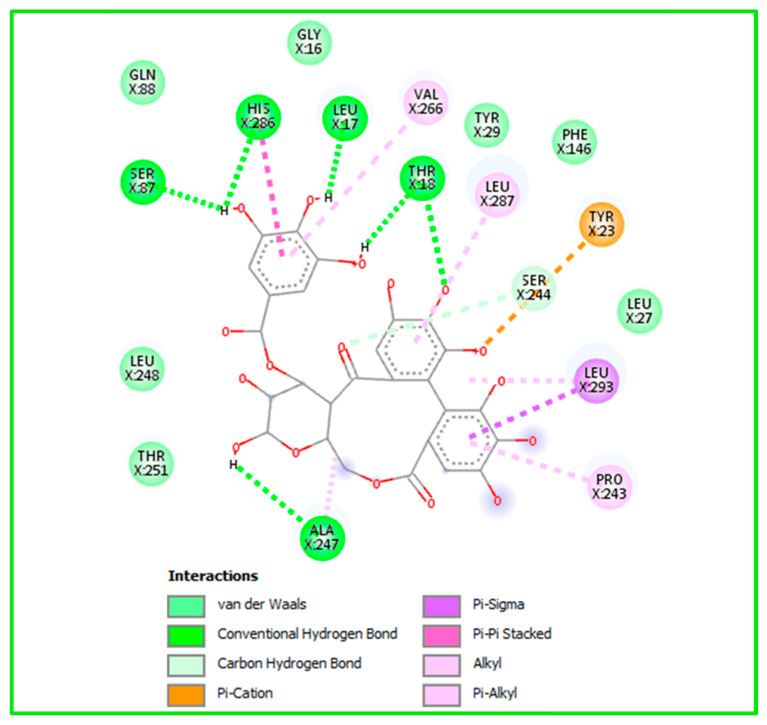
The most active molecule, HHDP-galloyl glucose, binds to the lipase binding cavity (PDB: 1ys1).

Regarding lipase, HHDP-galloyl glucose formed six hydrogen bonds between its hydroxyl groups and amino acids: Leu17, Thr18, Ser87, Ala247, and His286. In addition, a range of other interactions were identified, including carbon–hydrogen bonds (Ser244), Pi–cation (Tyr23), Pi–sigma (Leu293), Pi–Pi stacked (His286), and alkyl/alkyl (Pro243, Val266, Leu287, and Leu293), were also established (Figure 4).

**Figure 5:**
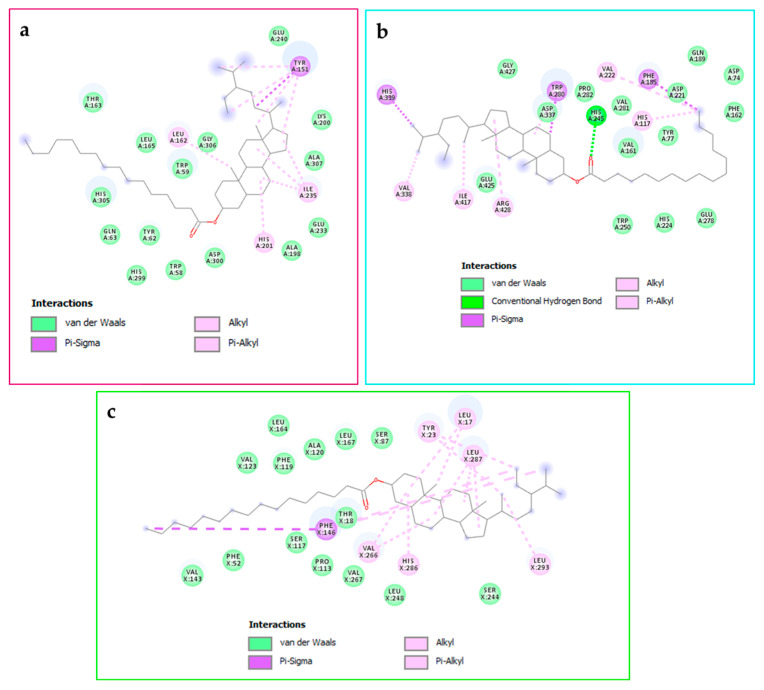
Binding modes of p-Coumaroyl hexose within the binding cavity of alpha-amylase (PDB:3baj) (**a**), alpha-glucosidase (PDB: 8yie) (**b**), and lipase (PDB: 1ys1) (**c**).

The second most active compound is p-coumaroyl hexose, which exhibited a strong inhibitory effect against α-amylase, α-glucosidase, and lipase with binding energy values of −7.6 and −8.1 kcal/mol, respectively. Interactions with α-amylase were observed through Pi–sigma (Tyr151), alkyl/Pi–alkyl (Tyr151, Leu162, and His201), and Ile235. Furthermore, the inhibitory effect against α-glucosidase involved H-bonds (His245), Pi–sigma (Phe185, Trp280, and His339) and alkyl/Pi–alkyl (His117, Val222, Trp280, Val338, Ile417, and Arg428) interactions. In addition, p-coumaroyl hexose was found to inhibit lipase through one Pi–sigma interaction (Phe146) and a multiple of alkyl/alkyl interactions (Leu17, Tyr23, Phe146, Val266, His286, Leu287, and Leu293) (Figure 5).

**Figure 6:**
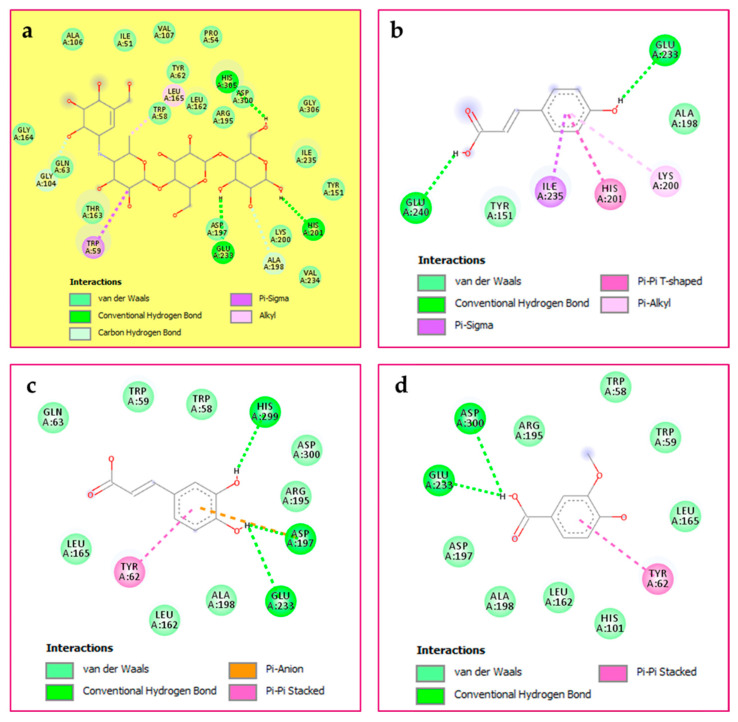
Acarbose (**a**), p-coumaric acid (**b**), caffeic acid (**c**), and vanillic acid (**d**) in the binding cavity of α-amylase (PDB: 3baj).

**Figure 7:**
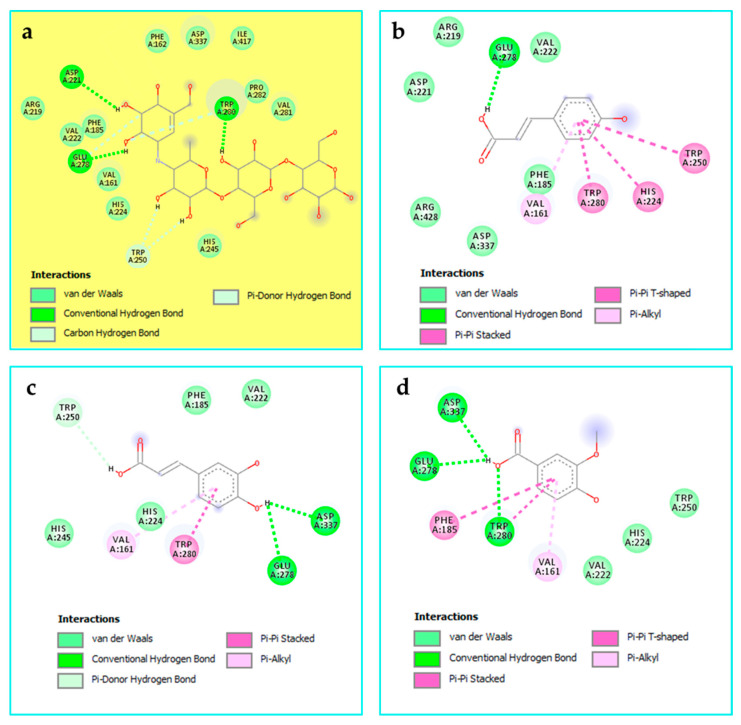
Acarbose (**a**), p-coumaric acid (**b**), caffeic acid (**c**), and vanillic acid (**d**) in the binding cavity of alpha-glucosidase (PDB: 8yie).

**Figure 8:**
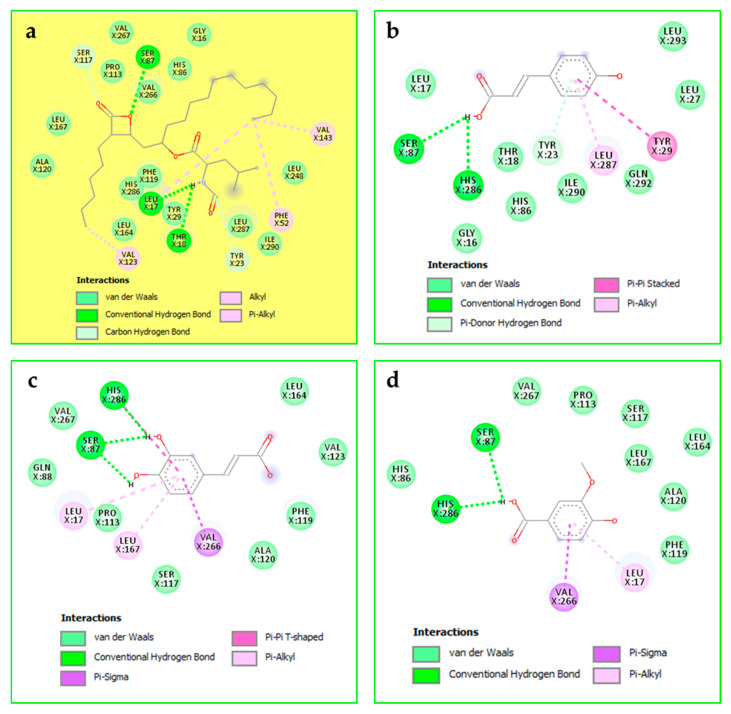
Compound binding modes: orlistat (**a**), p-coumaric acid (**b**), caffeic acid (**c**), and vanillic acid (**d**) in the lipase binding cavity (PDB: 1ys1).

The three remaining phenolic acids, p-coumaric acid, caffeic acid, and vanillic acid, exhibited enzyme-inhibitory effects against a-amylase, a-glucosidase, and lipase, although their activities were lower than those of the standards, acarbose and orlistat. The underlying mechanisms of these effects involved the formation of specific hydrogen bonds, Pi–alkyl, and Pi–Pi interactions (Figure 6, Figure 7 and Figure 8).

## 3. Materials and Methods

### 3.1. Plant Material

The quinoa seeds from 3 referenced accessions, R-132 (Acc. PI 478418; Potosi, Bolivia); 18 GR (Ames, 13724, USA), and DE-1 (Acc. PI 674266, Ecuador), were obtained from the USDA National Plant Germplasm System (NPGS) (Beltsville, MD, USA).

### 3.2. Plant Culture and Salt Treatment

Quinoa seeds were sown in 10 L plastic pots (10 seeds per pot) filled with refined sand and commercial peat and supplied with 50 mM (0.29%) (control), 150 mM (0.88%), and 200 mM (1.17%) NaCl, renewed daily. Pots were kept under greenhouse conditions at a natural photoperiod of 12 h/12 h (light/dark), a temperature of 20–30 °C, and a relative humidity level of 50–80%. At the flowering stage, the leaves were harvested, oven-dried at 45 °C for 72 h, then crushed, and stored at 4 °C until use.

### 3.3. Phytochemical Analyses

**Extract preparation**

Three salinity treatments were applied to quinoa plants grown in a greenhouse for this study: 50 mM (0.29%) (control), 150 mM (0.88%), and 200 mM (1.17%) NaCl. Through controlled irrigation, these salinity levels were gradually established while taking into account the frequency of watering and the plant’s water requirements, which were recalculated prior to each irrigation session. At the blossoming stage, leaves were collected and dried in an oven at 45 °C. To guarantee their acceptability for additional analysis, they were dried, crushed into a fine powder, and kept at 4 °C [27].

Using ethanol and water solvents, a multi-step procedure is used to extract phenolic chemicals from quinoa leaves during the blooming stage. To preserve the phenolic chemicals, fresh quinoa leaves are collected and allowed to air dry.

Dried quinoa leaf powder (100 g) was subjected to successive maceration at a solid-to-solvent ratio of 1:5 (*w*/*v*). Extraction was performed first with pure ethanol (96%), followed by distilled water, with several successive macerations until total exhaustion, where the final maceration cycle showed negligible coloration, indicating near-complete depletion of extractable phenolic compounds. The combined extracts from each solvent were filtered through Whatman No. 1 filter paper to remove solid residues and subsequently evaporated under reduced pressure using a rotary evaporator at 60 °C to obtain dry concentrates, which were stored at 4 °C for subsequent analysis.

**Total phenol content (TPC) determination**

The Folin–Ciocalteu technique was used to calculate the total phenolic content (TPC). In short, 500 µL of distilled water, 125 µL of 0.2 M Folin–Ciocalteu reagent, and 125 µL of quinoa leaf extract (1 mg/mL) were combined. Following mixing, 1250 µL of 7% Na_2_CO_3_ was added, and distilled water was used to get the volume down to 3 mL. After thoroughly homogenizing the mixture, it was incubated at 23 °C in the dark for 90 min. At 760 nm, absorbance was measured in comparison to a blank. The calibration curve was built using gallic acid (0–400 µg/mL), and the results were reported as mg gallic acid equivalents per 100 g of dry weight (mg GAE/100 g DW). Every analysis was carried out three times [35].

**Total flavonoid content (TFC) determination**

To put it briefly, 50 µL of 2% aluminum chloride solution (dissolved in methanol) was combined with 50 µL of quinoa leaf extract (1 mg/mL). Absorbance was measured at 405 nm after the mixture was incubated at room temperature for ten minutes. Milligrams of catechin equivalents (mg CAE/100 g dry weight) were used to express the flavonoid concentration [57].

**Condensed tannins (CT) determination**

With a few minor adjustments, the Sun et al. [58] method was used to determine condensed tannins. First, 1.5 mL of strong hydrochloric acid (36%), 3 mL of 4% vanillin solution (in methanol), and 500 µL of quinoa extract (1 mg/mL) were combined. At 500 nm, absorbance was measured following a 15 min incubation period. Milligrams of catechin equivalents (mg CAE/100 g dry weight) were used to express condensed tannins [58].

### 3.4. In Vitro Evaluation of Extract Bioactivities

**The 2,2-diphényl-1-picrylhydrazyle (DPPH)-radical scavenging assay**

The DPPH-radical scavenging activity was evaluated using the procedure described in reference [59]. Plotting the percentage of DPPH-radical scavenging activity against the extract concentration allowed for the determination of the concentration with 50% radical inhibition (IC50), represented as (µg/mL) [60]. One milliliter of the extract was mixed with 0.25 milliliter of DPPH at known quantities with a mixture of methanol. After giving the mixture a good shake, it was allowed to sit at room temperature in the dark for thirty minutes. The absorbance of the resultant solution was then measured at 517 nm to ascertain the values corresponding to the extracts’ capacity to convert the stable radical DPPH into the yellow-colored diphenylpicrylhydrazine. IC50 (µg/mL) values were used to express anti-radical activity, indicating the necessary extract dosages.

IC50 (µg/mL) values, which represent the extract dosages necessary to produce a 50% inhibition, were used to express anti-radical activity. The following formula was used to determine the capacity to scavenge the DPPH radical:

DPPH scavenging effect (%) = [(A0 − A1)/A0] ×100

where A0 is the control’s absorbance at 30 min and A1 is the sample’s absorbance at 30 min. Every sample was examined three times.

**Enzymatic activities evaluation**

**In vitro study**

- Amylase inhibitory activity: 150 µL of phosphate buffer (0.2 M, pH 6.8, 17 mM NaCl) was combined with 50 µL of the extract and 1.5 mg of soluble starch. After adding 10 µL of α-amylase, the mixture was incubated for 30 min at 37 °C. Next, 20 µL of NaOH and 20 µL of a reagent containing 44 µM 3,5-DNS, 106 µM potassium sodium tartrate tetrahydrate, and 40 µM NaOH were added to inhibit this reaction. For twenty minutes, the reaction mixture was incubated at 100 °C [61]. Acarbose was utilized as a reference when measuring the absorbance at 540 nm.
- Glucosidase inhibitory activity: 20 µL of α-glucosidase and 120 µL of phosphate buffer (0.2 M, pH 6.9) were combined with 10 µL of the extract. After that, 20 µL of 5 mM pNPG was added, and the mixture was incubated for 15 min at 37 °C. Then, 80 µL of a 0.2 M Na_2_CO_3_ was added to halt the reaction [62]. Acarbose was utilized as a reference when measuring the absorbance at 405 nm.
- Lipase inhibitory activity: After mixing 5 µL of the extract with 10 µL of 10 mg/mL lipase and 200 µL of Tris-HCl buffer (0.1 M, pH 8.5), the mixture was incubated at 37 °C for 25 min. Next, 5 µL of 5 mM 4-nitrophenyl caprate was added, and the mixture was incubated at 37 °C for an additional 15 min [63]. Orlistat was utilized as a reference when measuring the absorbance at 540 nm.

All enzyme inhibition assays were conducted under validated linear kinetic conditions. Absorbance measurements were confirmed to fall within the linear response range of each assay, and preliminary tests were performed to ensure proportionality between reaction rate and enzyme activity. Substrate concentrations were selected to be saturating (approximately 2–5 × K_m_, based on the literature values for α-amylase, α-glucosidase, and lipase), ensuring that the measured inhibition reflected enzyme–inhibitor interactions rather than substrate limitation.

### 3.5. Characterization of Phenolic Compounds by HPLC-PDA-ESI-MS/MS

An Agilent 1100 series HPLC system with a photodiode array detector (PDA) and a Micromass Autospec Ultima Pt triple quadrupole mass spectrometer with an ESI ion source running in the negative mode were used to identify the phenolic components of the most active extract. The mobile phases were acetonitrile (B) and acetic acid (0.1%) (A) at a flow rate of 0.25 mL/min. A Superspher^®^ 100 column (12.5 cm, 2 mm i.d., 4 µm particle size) kept at 45 °C was used to separate the different chemicals. The following linear gradient was used for elution: 0–5 min, 2% B; 5–75 min, 88% B; and 75–90 min, 2% B. The UV-Vis spectra in the 200–800 nm region and the full scan MS mode from 100 to 1000 amu were utilized. Capillary voltage 3.2 kV, cone voltage 115 V, probe temperature 350 °C, and ion source 110 °C were the ESI-MS settings. Phenolic compounds were tentatively identified by comparing their HPLC-PDA retention time, UV and mass spectra, and fragmentation patterns with those of commercial standards if available and/or the literature data [64].

### 3.6. In Silico Study

The Auto Dock vina program package was used to carry out molecular docking simulations [63]. The RSCB protein data bank (https://www.rcsb.org/) provided the structures of amylase (PDB ID: 3baj) [64], α-glucosidase (PDB ID: 8yie) [65] andlipase (PDB ID: 1ys1) [66], When the receptor input file was being prepared, the water molecules were first removed, and the missing hydrogen atoms and Gasteiger charges were then added to the system. All ligands and protein files were then prepared using AutoDock Tools 4.2 (PDBQT). For pdb: 3baj (x = 10.152, y = 15.838, and z = 41.117), pdb: 8yie (x = −1.874, y = −0.685, and z = 56.109), and pdb: 1ys1 (x = 13.241, y = 3.824, and z = 11.163), as well as with size_x = 30, size_y = 30, and size_z = 30. ACD (2017) (3D viewer) software (http://www.filefacts.com/acd3d-viewer-freeware-info (accessed on 31 August 2024)) was used to optimize all compound geometries, and Discovery Studio 2017R2 (https://www.3ds.com/products/biovia/discovery-studio (accessed on 31 August 2024)) was used to visualize and analyze interactions.

### 3.7. Statistical Analysis

The results are shown as mean ± standard error (SE), and each experiment was carried out in triplicate. Two-way ANOVA and Duncan’s multiple range post hoc test were used to determine statistical significance; differences were deemed significant at *p* < 0.05. SPSS software version 26.0 (SPSS Inc., Chicago, IL, USA) was used to analyze the data.

## 4. Conclusions

The results of the current study showed that salt treatment enhanced the TPC, TFC, and CT in the three quinoa accessions (18 GR, R-132, and DE-1) in a dose-dependent manner, thereby exerting a positive influence on their in vitro anti-radical and enzyme inhibitory effects against α-amylase, α-glucosidase, and lipase. A comparison of the accessions revealed that the DE-1 accession exhibited higher tolerance, characterized by preferential accumulation of ellagitannin HHDP-galloyl glucose and glycosylated phenolic acid p-coumaroyl hexose. The in silico study showed that the enzyme-inhibitory effect of both components was mediated through interaction types of hydrogen bond, Pi–cation, Pi–sigma, Pi–Pi stacked, and alkyl/alkyl.

These findings highlight quinoa as a promising crop for rehabilitating salt-affected environments and as a source of bioactive compounds with antioxidant, antidiabetic, and anti-obesity potential. Notably, the DE-1 accession under 200 mM NaCl emerges as a potent source of multitarget inhibitors of α-amylase, α-glucosidase, and lipase, supporting its translational potential for functional foods or phytopharmaceuticals. Enhancing the bioavailability of key compounds, particularly p-coumaroyl hexose and HHDP-galloyl glucose, through advanced delivery systems (e.g., nano-capsules, liposomes, and biodegradable polymers) represents a promising strategy. However, in vivo validation, including studies in streptozotocin-induced diabetic models, remains essential to confirm their therapeutic efficacy.

## Figures and Tables

**Table 1:** IC_50_ (µg/mL) of ethanol and water of the three accessions of quinoa leaf extracts in the DPPH test.

		Accessions			Standard
Solvents	Treatments (mM)	18 GR	R-132	DE-1	
**Ethanol**	50	230 ± 12 aA	120 ± 9 aB	118 ± 6 aB	BHT = 10.7 ± 0.61 ddc ddc
	150	120 ± 10 bA	90 ± 10 bB	110 ± 11 aAB	
	200	80 ± 14 cA	70 ± 6 cAB	60 ± 11 bB	
**Water**	50	30 ± 5 aA	19 ± 1 aB	11 ± 0.1 aC	
	150	28 ± 1 bA	16 ± 1 bB	8 ± 0.1 bC	
	200	26 ± 1 cA	14 ± 1 cB	6 ± 0.01 bC	

Values are means ± SE (n = 3). Lowercase and uppercase letters (a, b, c, d and A, B, C) indicate mean comparisons between treatments and accessions, respectively, based on Duncan’s test. Means followed by the same letter are not significantly different (*p* < 0.05). Red, green, and black colors indicate comparisons for ethanol between treatments and the standard for each accession; and blue, pink, and orange colors indicate comparisons for water between treatments and the standard for each accession.

**Table 2:** IC_50_ (µg/mL) of ethanol and water quinoa leaf extracts from the three accessions for amylase, glucosidase, and lipase.

Enzymes	Solvents	Treatments (mM)	Accessions			Standard
			18 GR	R-132	DE-1	
Lipase	Ethanol	50	185 ± 5 aA	161 ± 8 bA	104 ± 11 cA	Orlistat: 17.5 ± 2 CDD DDC
		150	155 ± 5 aB	125 ± 3 bB	70 ± 1 cC	
		200	150 ± 1 aB	114 ± 6 bC	77 ± 2 cB	
	Water	50	50 ± 3 abA	51 ± 5 aA	47 ± 4 bA	
		150	47 ± 2 aB	38 ± 5 bB	34 ± 3 cB	
		200	43 ± 4 aC	36 ± 4 bC	35 ± 4 cB	
α-amylase	Ethanol	50	160 ± 5 aA	150 ± 15 bA	151 ± 5 bA	Acarbose: 84.5 ± 4 CCB BCA
		150	140 ± 15 aB	120 ± 11 bB	100 ± 15 cB	
		200	130 ± 10 aB	132 ± 10 aB	90 ± 15 bB	
	Water	50	100 ± 5 bA	120 ± 20 aA	80 ± 15 cA	
		150	100 ± 15 aA	110 ± 10 aAB	60 ± 5 bB	
		200	90 ± 10 aA	100 ± 10 aB	62 ± 12 bB	
α-glucosidase	Ethanol	50	210 ± 20 aA	212 ± 10 aA	190 ± 11 bA	Acarbose: 92.5 ± 6.8 DDD CCC
		150	190 ± 10 aB	192 ± 8 aB	160 ± 7 bC	
		200	180 ± 5 aC	170 ± 6 bC	171 ± 2 bB	
	Water	50	160 ± 5 aA	158 ± 1 aA	159 ± 5 aA	
		150	150 ± 5 aB	140 ± 5 abB	130 ± 5 bB	
		200	151 ± 9 aB	142 ± 2 bB	120 ± 10 cB	

Values are means ± SE (n = 3). Lowercase and uppercase letters (a, b, c and A, B, C, D) indicate mean comparisons between treatments and accessions, respectively, based on Duncan’s test. Means followed by the same letter are not significantly different (*p* < 0.05). Red, green, and black colors indicate comparisons for ethanol between treatments and the standard for each accession; and blue, pink, and orange colors indicate comparisons for water between treatments and the standard for each accession.

**Table 3:** Profile of compounds identified by HPLC-PDA-ESI-MS/MS in quinoa leaf water extracts.

					Percentages (%)					
					18 GR		R-132		DE-1	
Tentative Identification	RT	Uv	[M-H]	Main Fragments	50 mM	200 mM	50 mM	200 mM	50 mM	200 mM
*p*-Coumaric acid	2.71	275	163	118	10.56 ± 0.4 aB	0.22 ± 0.02 bB	4.37 ± 0.22 bC	19.4 ± 0.6 aA	12.2 ± 0.26 aA	ND aC
Caffeic acid	3.21	322	179	135	11.45 ± 0.5 bC	28.51 ± 0.25 aB	40.9 ± 0.13 aA	34.16 ± 0.29 bA	30.26 ± 0.25 aB	ND aC
Citric acid	3.74	273	191	147	21.26 ± 0.8 aA	ND bB	7.3 ± 0.16 bB	8.56 ± 0.3 aA	5.03 ± 0.05 aC	ND aB
Vanillic acid	4.03	264	167	152	4.16 ± 0.05 bC	18.11 ± 0.45 aA	15.83 ± 0.05 aA	12.13 ± 0.05 bB	8.5 ± 0.01 aB	ND aC
Homovanillic acid	5.01	264	181	137	4.33 ± 0.01 aB	3.69 ± 0.01 bB	6.8 ± 0.01 aA	5.34 ± 0.05 bA	2.52 ± 0.026 aC	ND aC
Dihydroxybenzaldehyde glucuronide	13.62	274	313	137	2.42 ± 0.02 aB	0.69 ± 0.01 bB	1.6 ± 0.01 bC	2.01 ± 0.08 aA	2.66 ± 0.03 aA	ND aC
3-O-p-Coumaroylquinic acid	14.14	322	337	163	0.65 ± 0.01 aA	ND bA	ND aB	ND aA	ND aB	ND aA
Quercetin-3-O-galactosylrhamoside	17.54	254, 352	609	301	0.15 ± 0.005 bC	6.1 ± 0.05 aA	0.21 ± 0.01 bB	0.32 ± 0.01 aB	0.7 ± 0.01 aA	ND aC
Myricetin pentoside hexoside	17.72	350	611	317	0.3 ± 0.01 aB	0.17 ± 0.005 bB	0.14 ± 0.01 bC	0.76 ± 0.015 aA	0.63 ± 0.005 aA	ND aC
Quercetin-di-deoxyhexose	18.56	254, 352	593	301	0.25 ± 0.01 bA	1.51 ± 0.03 aA	0.17 ± 0.02 aB	ND bB	ND aC	ND aB
Methyl citrate	18.74	-	205	111	0.31 ± 0.01 bA	1.29 ± 0.01 aA	0.29 ± 0.01 aA	ND bB	ND aB	ND aB
Rutin	18.81	254, 354	609	301	1.56 ± 0.01 bA	10.8 ± 0.015 aA	0.21 ± 0.01 bC	1.24 ± 0.02 aB	0.51 ± 0.015 aB	ND aC
Kaempferol dihexoside	19.78	250, 352	609	285	1.09 ± 0.005 bB	4.51 ± 0.02 aA	ND bC	0.72 ± 0.02 aB	1.21 ± 0.01 aA	ND aC
Quercetin hexoside	21.46	254, 354	463	301	0.065 ± 0.005 aB	ND bA	8.59 ± 0.015 aA	ND bA	ND aC	ND aA
*p*-coumaroyl hexose	32.08	275	519	325, 163	1.91 ± 0.09 aB	0.38 ± 0.015 bC	1.4 ± 0.015 bC	5.99 ± 0.05 aB	3.48 ± 0.02 bA	24.16 ± 0.03 aA
Hhdp-galloyl glucose	33.65	254	633	463, 301	1.14 ± 0.02 bA	9.51 ± 0.04 aA	0.17 ± 0.01 bC	0.51 ± 0.01 aC	0.42 ± 0.02 bB	4.3 ± 0.15 aB
Protocatechuic acid hexose	39.21	275	329	153	0.38 ± 0.02 aA	0.41 ± 0.02 aA	ND aB	ND aB	ND aB	ND aB
Isosakuranetin pentoside rutinoside	48.18	-	725	285	0.1 ± 0.007 aB	ND bB	0.66 ± 0.001 aA	0.11 ± 0.007 bA	ND aC	ND aB

Values are means ± SE (n = 3). Lowercase and uppercase letters (a, b and A, B, C) indicate mean comparisons between treatments and accessions, respectively, based on Duncan’s test. Means followed by the same letter are not significantly different (*p* < 0.05). Percentages (%) represent relative peak-area values normalized against the total photodiode array (PDA) signal recorded over 200–800 nm, and do not correspond to absolute concentrations (e.g., mg/g DW). Compound identification is considered tentative and was based on comparison of retention times, UV spectra, precursor ions, and MS/MS fragmentation patterns with available commercial standards (for simple phenolic acids) and/or published literature data (for glycosylated and complex derivatives). Semi-quantitative analysis was performed without external calibration; therefore, no calibration curves or LOD/LOQ values are provided. ND: not detected.

**Table 4:** Binding energy of the docked compounds in the binding cavity of α-amylase (PDB:3baj), α-glucosidase (8yie), and lipase (1ys1).

Compound	Binding Energy (kcal/mol)		
	α-Amylase	α-Glucosidase	Lipase
p-Coumaric acid	−4.7	−5.2	−5.8
Caffeic acid	−6.2	−5.8	−5.4
Vanillic acid	−4.9	−4.8	−5.2
p-Coumaroyl hexose	−7.6	−7.6	−8.1
HHDP-galloyl glucose	−10.5	−9.5	−9.8
Acarbose	−7.4	−6.3	-
Orlistat	-	-	−6.3

## Data Availability

The original contributions presented in this study are included in the article. Further inquiries can be directed to the corresponding authors.
